# Molecular phylogeny reveals a new species of ghost electric knifefish *Porotergus* Ellis 1912 (Gymnotiformes: Apteronotidae), from the Amazon basin

**DOI:** 10.1111/jfb.70085

**Published:** 2025-07-14

**Authors:** Marina B. Mendonça, Luiz A. W. Peixoto, Carine C. Chamon, A. Akama, C. David de Santana

**Affiliations:** ^1^ Laboratório de Ictiologia de Soure Universidade Federal do Pará, Campus Soure Soure Brazil; ^2^ Instituto de Estudos Costeiros de Bragança Universidade Federal do Pará Bragança Brazil; ^3^ Laboratório de Ictiologia Sistemática, Núcleo de Estudos Ambientais Universidade Federal do Tocantins, Campus de Porto Nacional Tocantins Brazil; ^4^ Coordenação de Zoologia Museu Paraense Emílio Goeldi Belém Brazil; ^5^ National Museum of Natural History Department of Fishes Washington District of Columbia USA

**Keywords:** DNA barcode, electric fish, ichthyofauna, Neotropics, taxonomy

## Abstract

A new species of ghost electric knifefish, *Porotergus sambaibensis* sp. nov., is described from the Javaés River, a tributary of the Araguaia River in Brazil. The new species was assigned to the genus *Porotergus* as the closest relative to *Porotergus gimbeli* through maximum likelihood reconstruction of a concatenated multilocus dataset. Additionally, the origin of *adductor mandibulae, pars stegalis* in *P*. *sambaibensis* sp. nov. provided further evidence to support the molecular hypothesis. External and internal anatomical characters diagnosed the new species. DNA barcode data were used to test species monophyly and its genetic divergence from other species in the clade. *Porotergus sambaibensis* sp. nov. is distinguished from its closely related species by the colour pattern of the trunk, dark brown; the lower count of total anal‐fin rays, 146–160; the higher number of teeth rows on the dentary, two; the presence of premaxilla teeth; two prominent foramina on dorsal portion of hyomandibula and its distribution pattern; and the second basibranchial, unossified. The genetic divergence between the new species and its relatives ranged from 3.7% in *P. gimbeli* to 10.3% in *Porotergus duende*. The species was categorised as deficient data (DD) based on the International Union for Conservation of Nature (IUCN) criteria.

## 1 INTRODUCTION

The Apteronotidae is the most species‐rich family in Gymnotiformes, containing over 95 valid species within 15 genera, and is found in areas ranging from eastern Panama to southeast Argentina (Bernt et al., 2019; Fricke et al., 2024). Apteronotids are commonly found in the river channels of the Amazon and Orinoco lowlands (Cox‐Fernandes et al., 2004; Crampton, 2007, 2011; Marrero & Taphorn, 1991) where they use their electric organ discharge to communicate and hunt, with frequencies ranging from 800 Hz in *Orthosternarchus* to 2300 Hz in *Sternarchella* (Crampton, 2007).

In recent years, there have been various attempts to establish relationships between different hierarchical levels among apteronotids using both morphological and molecular data (Albert, 2001; Bernt et al., 2018, 2019; Ivanyisky & Albert, 2014; Peixoto & de Pinna, 2022; Peixoto et al., 2019, 2022; Tagliacollo et al., 2016a; Triques, 2005), as in the case of the ‘Navajini’ (invalid name: ICZN, 1999: Art. 11.7.1.1; Ferraris et al., 2017) comprising *Adontosternarchus* Ellis, 1912; *Compsaraia* Albert, 2001; *Porotergus* Ellis, 1912; *Sternarchella, Sternarchogiton* Eigenmann, 1905; and *Tenebrosternarchus* Bernt et al., 2020. Since then, there have been a few updates to the apteronotids taxonomic nomenclature, such as those made by Evans et al. (2017) and Bernt et al. (2020). However, further changes will be needed to ensure the current understanding of phylogenetic relationships within Apteronotidae. For instance, recent studies recovered ‘*Apteronotus*’ *bonapartii* species group [i.e., ‘*A*’. *bonapartii* (Castelnau, 1855) from the lowland Amazon basin, ‘*Apteronotus apurensis’* Fernández‐Yépez, 1968 from Orinoco basin and ‘*Apteronotus ellisi’* (Arámburu, 1957) from the Paraná River] inserted within *Porotergus* (Bernt et al., 2018, 2019; Tagliacollo et al., 2016b).

*Porotergus* was erected by Ellis (1912) to include *Porotergus gymnotus* from the Essequibo basin and *Porotergus gimbeli* Ellis, 1912 from the Amazon lowlands. The genus was defined by the absence of scales on the dorsal region of the body anterior to the dorsal mid‐sagittal electroreceptive filament; the presence of large scales above the lateral line; the presence of teeth in both jaws; and a long mouth cleft, reaching or passing the vertical through the anterior margin of the eye. De Santana and Crampton (2010), in the taxonomic review of *Porotergus*, described *Porotergus duende* from the western Amazon basin and provided a few additional characteristics to differ *Porotergus* from the remaining apteronotids, which are as follows: the absence of a pale stripe on the head; the poorly ossified infraorbital series; and a maxilla without an anterior shelf. Recently, Peixoto and de Pinna (2022) proposed the origin of *adductor mandibulae and pars stegalis*, not including the sphenotic and pterosphenoid as synapomorphies for the genus (Peixoto & de Pinna, 2022: Ch. 18, 21, respectively).

Conversely, the phylogenetic position of *Porotergus* was recently scrutinised by several authors. For instance, Tagliacollo et al. (2016a), using a model‐based total evidence approach in a dataset comprising six genes and 223 morphological characters for 34 apteronotid genera, recovered the monophyly of *Porotergus* + ‘*A*.’ *bonapartii* species group, a hypothesis corroborated by subsequent authors (e.g., Peixoto et al., 2022: fig. 9). In contrast, anatomy‐based studies recovered *Porotergus* as the sister group of *Tenebrosternarchus* + *Sternarchogiton* (Peixoto et al., 2019; Peixoto & de Pinna, 2022).

During a scientific expedition to survey the ichthyofauna of the Araguaia basin in Brazil, several specimens of an undescribed apteronotid were collected on the rocky bank alongside the Javaés River. Based on a multilocus dataset, we built a hypothesis for its phylogenetic relationships and then proposed it as a new species based on morphological and molecular evidence.

## 2 MATERIALS AND METHODS

### 2.1 Collecting samples

Specimens were found living inside the rock crevices. To collect them, rocks were extracted from the riverbed, broken with a hammer and pickaxe. The specimens were then manually extracted and immediately placed in a solution of clove oil. **Nomenclature**. Our use of *Porotergus* is related to the ‘Navajini A’ (Bernt et al., 2019: 301: 4.1 New classification; pg. 305: Figure 6), which includes ‘*A*.’ *apurensis*, ‘*A*’. *bonapartii* and ‘*A*’. *ellisi*. **Abbreviations**. Institutional acronyms are under Fricke and Eschmeyer (2024). Other abbreviations in the text are μCT scan = microcomputed tomography. **Morphological analyses**. Data were taken from the left side of all specimens with the aid of a stereomicroscope and digital calliper with 0.1 mm of precision. Measurements, meristic data and bone terminology follow Peixoto et al. (2021). The measurements are presented as proportions of length to the end of the anal fin (LEA), except for subunits of the head, which are given as proportions of head length (HL). Counts of fin rays are presented in lowercase Roman numerals (unbranched rays) and Arabic numerals (branched rays). Anal‐fin ray counts were only taken from intact specimens. After each count, the frequencies are provided in parentheses, with an asterisk denoting the counts for the holotype. Parameters for radiographs and X‐ray microcomputed tomography (CT scan) are provided by Peixoto et al. (2019). Two specimens were cleared and stained according to Taylor and van Dyke (1985). For additional examined specimens and cleared and counterstained specimens, see de Santana and Crampton (2007), de Santana and Crampton (2010); de Santana and Vari (2010, 2013); Peixoto et al. (2021, 2022). **DNA extraction, amplification and sequencing**. Amplification protocol followed Tagliacollo et al. (2016a). DNA extraction, amplification and sequencing were realised following standard procedures at the Molecular Biology Laboratory of the Museu Paraense Emílio Goeldi (Belém, Pará, Brazil) and the Laboratories of Analytical Biology of the National Museum of Natural History. All obtained sequences were deposited in GenBank (Table 1). **Alignments, molecular delimitation and phylogenetic analyses**. Raw sequences were edited in the CodonCode Aligner (www.codoncode.com) and manually aligned.

**TABLE 1:** List of species used in phylogenetic analysis.

Species	GenBank *COI*	GenBank *Cytb*	GenBank *16S*	GenBank *RAG2*	Voucher	Tissue number
*Porotergus sambaibensis*	PV828398	NA	NA	NA	MPEG 34487_1	504
*P. sambaibensis*	PV828399	NA	NA	NA	MPEG 34487_2	505
*P. sambaibensis*	NA	PV853937	PV828130	NA	MPEG 39641_1	AP1
*P. sambaibensis*	NA	PV853938	NA	PV853932	MPEG 39641_2	AP2
*P. sambaibensis*	NA	PV853939	PV828131	PV853933	MPEG 39641_3	AP3
*P. sambaibensis*	NA	PV853940	PV828132	PV853934	MPEG 39641_4	AP4
*P. sambaibensis*	NA	NA	PV828133	PV853935	MPEG 39641_5	AP5
*P. sambaibensis*	NA	PV853941	NA	PV853936	MPEG 39641_6	AP6
*Porotergus duende*	MK401925	MK389120	MK382660	MK389020	MUSM 54676	IQ02080115
*P. duende*	MG653356				ANSP 200463	A083
*P. duende*	MG653357				ANSP 200463	A086
*P. duende*	ON303423				MCP 37359	
*P. duende*	MK401914	MK389107	MK382647		ANSP 200483	A092
*Porotergus gymnotus*	MK401918	MK389111	MK382651	MK389013	ANSP 189647	
*P. gymnotus*	MZ050984				MHNG uncat	GF15‐513
*P. gymnotus*	MZ051138				MHNG uncat	GF15‐512
*P. gymnotus*	MZ051563				MHNG uncat	GF15‐510
*Porotergus gimbeli*	MG653383	MG653334	MG653285	MG653426	ANSP 200375	SA015
*P. gimbeli*	MK401953				ANSP 200375	SA013
*P. gimbeli*	MK401961	MK389158	MK382701	MK389056	ANSP 200407	SA052
*P. gimbeli*					MUSM 54677	IQ03100115
*P. gimbeli*	ON303424				MCP 37529	
*Apteronotus apurensis*	MK401986	MK389181	MK382726	MK389076	ANSP 198350	T4861
*A. apurensis*	MG653396				ANSP 198404	T4653
*A. apurensis*	MG653399	MG653350	MG653301	MG653441	ANSP 198385	T4734
*Apteronotus ellisi*	MK401991				MNHNP 4173	T248
*A. ellisi*	MK401998	MK389193	MK382738		MNHNP 4173	T297
*A. ellisi*	KR491550	KR491674	KR260194	KR491838	LBP 24040	24,040
*Apteronotus bonapartii*	KR491551	KR491673	KR260191	KR491835	ANSP 182585	
*A. bonapartii*		MG653321	MG653272	MG653414	MUSM 54641	IQ05070714
*A. bonapartii*	MG653378				MUSM 54646	IQ10070714
*A. bonapartii*	ON303330				ZUEC 17150	
*A. bonapartii*	MK401950				MUSM 37171a	
*A. bonapartii*	KR491549				MUSM 37171	
*A. bonapartii*	MK401951				MUSM 37171b	
*Tenebrosternarchus preto*	MG653364	MG653315	MG653266	MG653408	MUSM 54617	IQ01040714c
*Sternarchella schotti*	MK401930	MK389126	MK382666	MK389026	MUSM 54678	IQ07120115
*Compsaraia samueli*	MG653359	MG653310	MG653261	MG653405	ANSP 200455	A091
*Melanosternarchus amaru*	MG653391	MG653342	MG653293	MG653433	LSUMZ 20732	SA060
*Orthosternarchus tamandua*	MK401966	MK389163	MK382706	MK389060	ANSP 200440	SA072
*Sternarchorhamphus muelleri*	MK401965	MK389162	MK382705	MK389059	ANSP 200431	SA064
*Megadontognathus kaitukaensis*	MK401919	MK389112	MK382652	MK389014	ANSP 195961	
*Sternarchorhynchus mormyrus*	MG653380	MG653331	MG653282	MG653424	MUSM 54650	IQ14070714
*Sternarchorhynchus galibi*	KR491558	KR491656	KR260179	NA	ANSP 187155	
*Sternarchorhynchus roseni*	MK401981	MK389176	MK382721	MK389071	ANSP 198343	T4837
*Sternarchella rex*	MK402005	MK389200	MK382745	MK389090	ANSP 200271	SA224
*Sternarchella calhamazon*	MG653390	MG653341	MG653292	MG653432	ANSP 200394	SA043
*Pariosternarchus amazonensis*	MG653373	MG653324	MG653275	MG653417	ANSP 200454	IQ06100115
*P. amazonensis*	MK402014				FMNH 128402	SA300
*Sternarchogiton porcinum*	MG653402	MG653353	MG653304	MG653444	ANSP 198347	T4851
*Sternarchogiton nattereri*	MG653385	MG653336	MG653287	MG653427	ANSP 200393	SA025
*Apteronotus rostratus*	MK401970	MK389166	MK382710	MK389063	STRI 24821	
*Platyurosternarchus macrostoma*	MK401960	MK389157	MK382700	MK389055	ANSP 200406	SA048
*Apteronotus eschmeyeri*	MK402003	MK389198	MK382743	MK389088	ANSP	T13345
*Apteronotus mariae*	MK402001	MK389196	MK382741	MK389086	ANSP	T13321
*Apteronotus albifrons*	MG653400	MG653351	MG653302	MG653442	ANSP 198394	T4771
*Adontosternarchus balaenops*	KR491528	KR491637	KR260164	KR491809	UFRGS 14826	182,572
*Adontosternarchus devenanzii*	MG653394	MG653345	MG653296	MG653436	ANSP 198405	T4651
*Adontosternarchus nebulosus*	MK402009	MK389204	MK382749	MK389094	ANSP 200311	SA281
*Apteronotus cuchillejo*	MK401972	MK389168	MK382712	NA	ANSP	T14276
*Parapteronotus hasemani*	MK401916	MK389109	MK382649	MK389011	ANSP 200498	A107
*Apteronotus acidops*	MK401989	MK389184	MK382729	MK389078	MNHNP 4175	T532

Genetic information from six individuals was gathered to determine the position of the new taxon in *Porotergus*. This included fragments of the mitochondrial genes *COI* (cytochrome oxidase subunit I, 673 bp), *Cytb* (cytochrome b, 1049 bp) and *16S* (16S rRNA, 527 bp), as well as one nuclear gene *RAG2* (recombination activating gene 2, 697 bp). Partitioned genes were concatenated dataset in a single matrix (totalling 2946 bp), including sequences from all six *Porotergus* species (‘*A*’. *apurensis*, ‘*A*’. *bonapartii*, ‘*A*’. *ellisi, P. duende, P. gimbeli* and *P. gymnotus*), as well as sequences from one individual of 26 out‐group species (representing all 16 valid apteronotid genera, as listed in Ferraris et al., 2017, Table 2). A maximum likelihood (ML) search and slow bootstrapping (1000 replicates) under GTRGAMMA model were conducted to infer the phylogenetic relationships of the new taxon using randomised accelerated ML – RAxML‐NG 1.2.0 (Kozlov et al., 2019) in CIPRES (Miller et al., 2010). The tree was rooted a posteriori in *Orthosternarchus* + *Sternarchorhamphus*, the hypothesised sister clade for all remaining apteronotids (Albert, 2001; Albert & Campos‐da‐Paz, 1998; Bernt et al., 2019; Tagliacollo et al., 2016a). Two individuals belonging to the new species were barcoded for *COI*. Bayesian inference (BI) under a strict clock and coalescent model in BEAST2* 2.4.8 (Bouckaert et al., 2014) for an Markov chain Monte Carlo run of 10 million generations under the TIM2GAMMA model were used to test the reciprocal monophyly of each species. To examine chain convergence, trace files were used and considered adequate when the effective sample size (ESS) for all parameters was over 200. The visualisation was made using Tracer version 1.7.2 (Rambaut et al., 2018). Posterior probability was used to infer node supports. Using the Kimura two parameter (K2P) in MEGA 6.06, pair‐wise genetic distances were calculated for 27 vouchered *COI* sequences (length of 665 bp) belonging to all seven species in the in‐group clade. *Pariosternarchus amazonensis* and *Sternarchella rex* were used as out‐groups. Trees were visualised and edited using the FigTree version 1.4.4 (Rambaut, 2018). **Ethical statement**. This study used only ethanol‐preserved specimens deposited in collections and did not involve experimentations or examinations.

**TABLE 2:** Morphometric data of *Porotergus sambaibensis*. *N* = 15.

	Holotype	Range	Mean	SD
Total length (mm)	110.2	46–110.2	70.8	17.3
Length to the end of the anal fin (LEA) (mm)	91.6	39.5–91.6	59.6	14.1
Head length (mm)	14.6	7.4–14.6	10.3	2.1
% LEA				
Head length (HL)	16.0	15.8–19.2	17.4	1.2
Pre‐anal distance	15.0	15–19.7	17.0	1.5
Pre‐pectoral distance	18.1	18–21	19.5	1.0
Snout to anus distance	9.8	9.6–14.9	12.2	1.6
Body depth at pectoral fin	13.3	13.1–16.3	14.5	0.9
Body depth at anal fin	11.9	11.9–16	13.5	1.2
Body width	4.3	4.3–6.3	5.4	0.5
Anal‐fin length	85.3	4–6.3	89.3	8.0
Pectoral‐fin length	9.6	8.7–11.5	10.3	0.8
Tail length	22.4	15.3–22.4	18.7	2.3
Caudal‐fin length	4.8	4–6.3	5.3	0.6
Dorsal electric organ	41.9	41.9–44.9	6.2	15.8
% HL				
Snout length	33.1	27.1–97.6	36.1	17.1
Internasal distance	11.7	8.6–14.2	11.8	1.5
Snout to posterior naris distance	22.6	21.9–31.9	25.8	2.8
Posterior naris to orbit distance	8.0	3.7–8	5.6	1.3
Internarial width	9.6	8.9–13.6	11.5	1.3
Orbital diameter	11.1	11.1–16.2	13.6	1.4
Postorbital distance	58.3	54.7–63.3	59.5	2.7
Opercular opening	19.8	12.3–20.6	18.1	2.2
Suborbital depth	28.1	25.5–29.9	28.2	1.5
Interorbital distance	24.2	23–31	25.7	2.1
Head width at opercle	40.0	36–42.2	38.7	2.2
Head width at orbits	33.1	25.7–39.1	32.2	2.9
Head depth at supraoccipital	65.8	65.8–82.1	75.0	5.2
Head depth at orbits	48.4	46–58.6	52.0	4.0
Maxilla length	47.2	43.1–52.1	47.2	2.8
Oral width	10.7	10.7–15.5	13.4	1.8
% Tail length				
Caudal filament depth	2.0	1.7–2.5	2.1	0.3

Abbreviation: SD, standard deviation.

## 3 RESULTS

### 3.1 Monophyly and phylogenetic relationships within *Porotergus*

**FIGURE 1:**
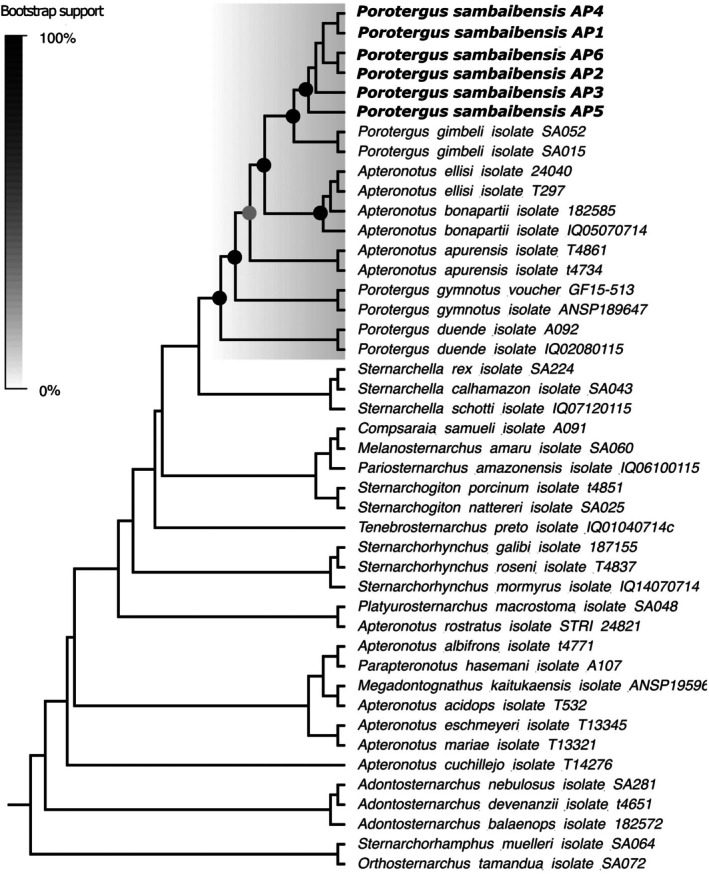
Phylogeny of species in *Porotergus* (grey) from maximum likelihood reconstruction of the concatenated matrix for 1000 bootstrap replicates. A topology with bootstrap labels for all Apteronotidae nodes is presented in Supplemental Figure S1.

**FIGURE 2:**
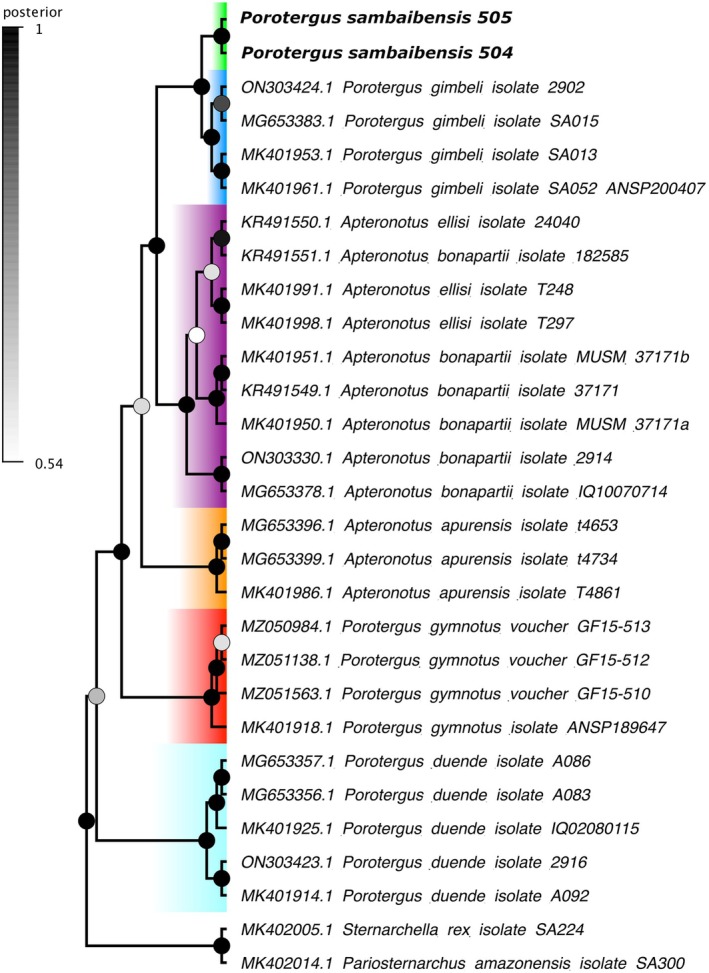
Phylogeny of species in *Porotergus* based on Bayesian inference of cytochrome oxidase subunit (*COI*) matrix with posterior probability for branches and nodes. Green: *Porotergus sambaibensis*; dark blue: *Porotergus gimbeli*; purple: ‘*Apteronotus*’ *bonapartii* + ‘*Apteronotus*’ *ellisi*; orange: ‘*Apteronotus*’ *apurensis*; red: *Porotergus gymnotus*; light blue: *Porotergus duende*.

The ML corroborated with strong support from bootstrap, the monophyly of *Porotergus* with ‘*A*’. *bonapartii* species group inserted within this clade. In *Porotergus*, it recovered *P*. *duende* from the western Amazon as the sister to all other species within *Porotergus*. In consecutive order, *P. gymnotus* from the Essequibo and Maroni basins, ‘*A*’. *apurensis*, a clade with ‘*A*’. *bonapartii* from the lowland Amazon basin and ‘*A*’. *ellisi* from the Paraná River and a clade including *P. gimbeli* from the Amazon lowland and the new species from the Araguaia basin formed sister relationships. In contrast to all nodes within *Porotergus*, which had a bootstrap support of over 98%, ‘*A*’. *apurensis* node was low at 60% (Figures 1 and 2).

### 3.2 *Porotergus sambaibensis*, new species

urn:lsid:zoobank.org:act:12CFFA38‐5F4A‐4CC9‐B60C‐70727F7CCE6F.

### 3.3 Holotype

MPEG 39639, 110.2 mm TL, 91.6 mm LEA, Brazil, Tocantins, Pium, Javaés River at pedral da Sambaíba, 10°00′00.6″ S 50°01′28.3″ W; Chamon, C., Pereira, T., Fernandes, A., Oliveira, E.; 16 September 2017 (ex UNT 17380).

### 3.4 Paratypes

All from Brazil, Tocantins, Pium, Javaés River at pedral da Sambaíba, 10°00′00.6″ S 50°01′28.3″ W: INPA‐ICT 060674, 5, 60.8–86.5 mm LEA; MZUSP 129865, 2 μCT, 86.2–98.6 mm LEA [all collected with the holotype (ex UNT 17380)]; MPEG 39640, 6, 42.3–56.8 mm LEA, 03 October 2018, Mendonça, M. B.; UNT 15053, 4, 72.4–88.8 mm LEA, 18 May 2016, Akama, A., Chamon, C. Mendonça, M. B.; UNT 15679, 7, 51.8–57.4 mm LEA, 02 November 2017, Chamon, C., Krolow, T., Oliveira, E., Bezerra, C., Pereira, T.; UNT 17380, 5, 46–87.8 mm LEA, 1 CS, 87.8 mm LEA, collected with the holotype; UNT 17407, 14 77.4–90.2 mm LEA, 16 September 2017, Chamon, C., Pereira, T., Fernandes, A., Oliveira, E.

### 3.5 Diagnosis

*P. sambaibensis* can be distinguished from *P. duende* by the colour pattern of the trunk, dark brown (vs. light brown to pale straw pigmentation). The new species can be distinguished from ‘*A*’. *apurensis* and *P*. *gimbeli* by the lower count of total anal‐fin rays, 146–160 (vs. 171–198). *P. sambaibensis* can be further distinguished from ‘*A*’. *bonapartii* by the premaxilla dentition pattern, five teeth arranged in two irregular rows (vs. three tooth rows, each with 2–4 teeth); by the lateral ethmoid position, straight (Figure 4; vs. the lateral ethmoid obliquely positioned, extending ventrally from the frontal at an angle towards the dorsal surface of the parasphenoid; Hilton & Cox Fernandes, 2017: Figure 4; Peixoto & de Pinna, 2022: fig. 25); by the presence of two foramina on the dorsal portion of hyomandibula (Figure 6 vs. one; Hilton & Cox Fernandes, 2017: Figure 4). The new species can be distinguished from *P. gimbeli* by the unelaborated chin (vs. prominent swelling on the chin, e.g., de Santana & Crampton, 2010: Figure 4). It can be further differentiated from *P. duende* by the higher number of teeth rows on dentary, two (vs. one); by the presence of premaxilla teeth (vs. absent); and by the second basibranchial, unossified (vs. ossified). *P. sambaibensis* is differentiated from *P. gymnotus* by the number of premaxillary teeth, five (vs. two). The new species can be distinguished from ‘*A*’. *ellisi* by the anal‐fin rays, 146–160 (vs. 170–190).

### 3.6 Species reciprocal monophyly and molecular divergencies

BI *COI* reconstruction recovered six distinct and well‐supported species identified, except for ‘*A*’. *ellisi* that emerged nested in ‘*A*’. *bonapartii* (Figure 2; Bernt et al., 2019). When compared to its close relatives, the new species displayed a 3.7% divergence from *P. gimbeli*, 6.9% from ‘*A*’. *bonapartii*, 7% from ‘*A*’. *ellisi*, 8.8% from ‘*A*’. *apurensis*, 8.9% from *P. gymnotus* and 10.3% from *P. duende* (Table 3).

**TABLE 3:** Percentage of genetic divergence, Kimura two‐parameter model, in the cytochrome oxidase subunit (*COI*) gene among seven species in *Porotergus*.

	*Porotergus sambaibensis*	*Porotergus gimbeli*	*‘Apteronotus’ bonapartii*	*‘Apteronotus’ ellisi*	*‘Apteronotus’ apurensis*	*Porotergus gymnotus*	*Porotergus duende*
*P. sambaibensis*							
*P. gimbeli*	3.7						
*‘A’. bonapartii*	6.9	6.5					
*‘A’. ellisi*	7	6.4	0.8				
*‘A’. apurensis*	8.8	9.8	7.1	7.1			
*P. gymnotus*	8.9	10	7.5	7.7	6.5		
*P. duende*	10.3	12.9	10.6	10.6	9.3	11	

### 3.7 Description

**FIGURE 3:**
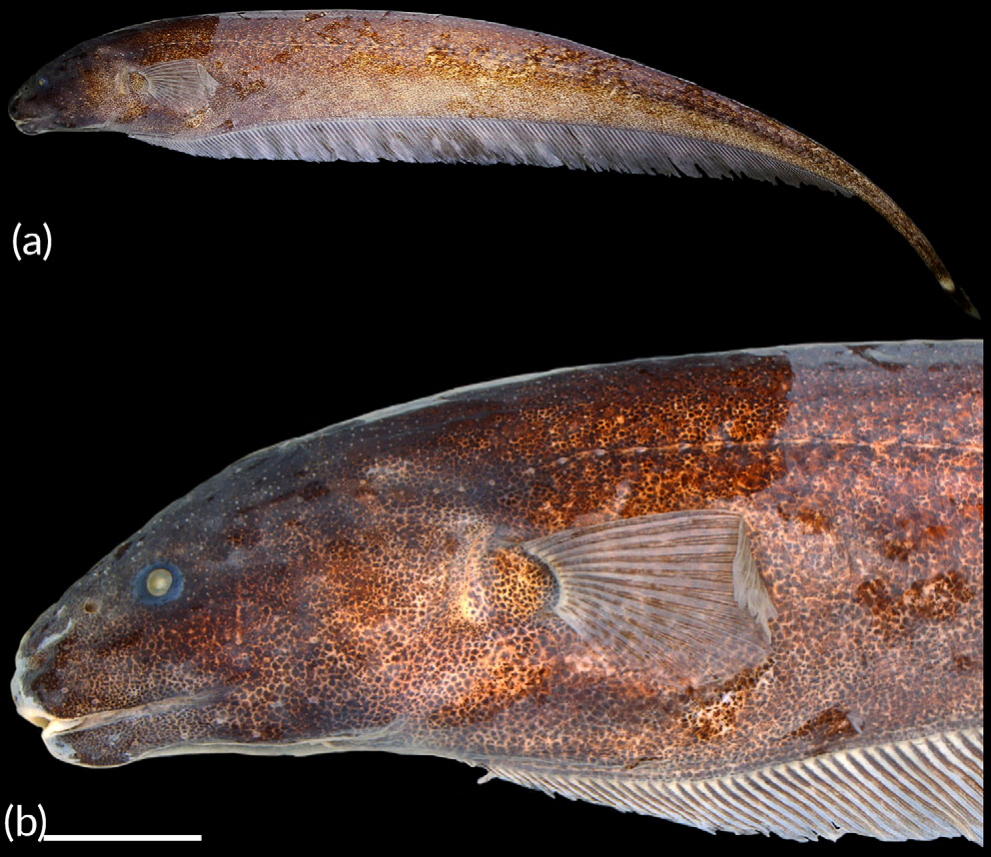
*Porotergus sambaibensis*, holotype, MPEG 39639, 110.02 mm total length (TL). (a) Full body; (b) head. Scale bar = 5 mm.

**FIGURE 4:**
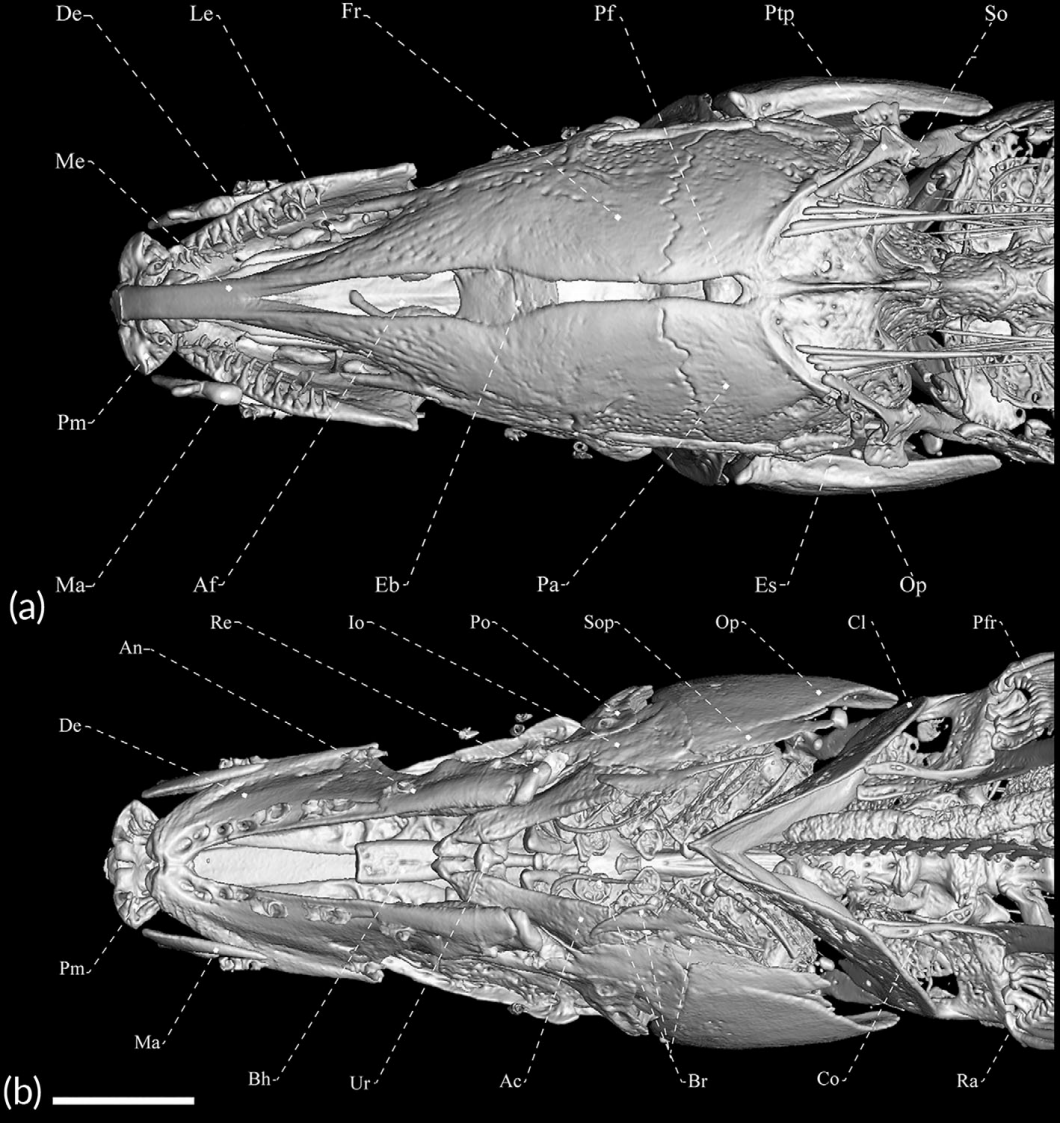
Dorsal view of the skull of *Porotergus sambaibensis*, paratype, MZUSP 129865, 98.6 mm length to the end of the anal fin (LEA). (a) Dorsal view; (b) ventral view. Scale bar: 2.5 mm.

**FIGURE 5:**
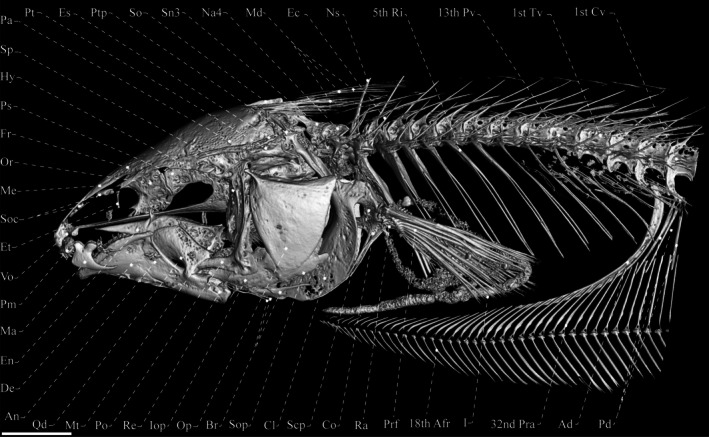
Lateral view of skull of *Porotergus sambaibensis*, paratype, MZUSP 129865, 98.6 mm length to the end of the anal fin (LEA). Scale bar: 3 mm.

**FIGURE 6:**
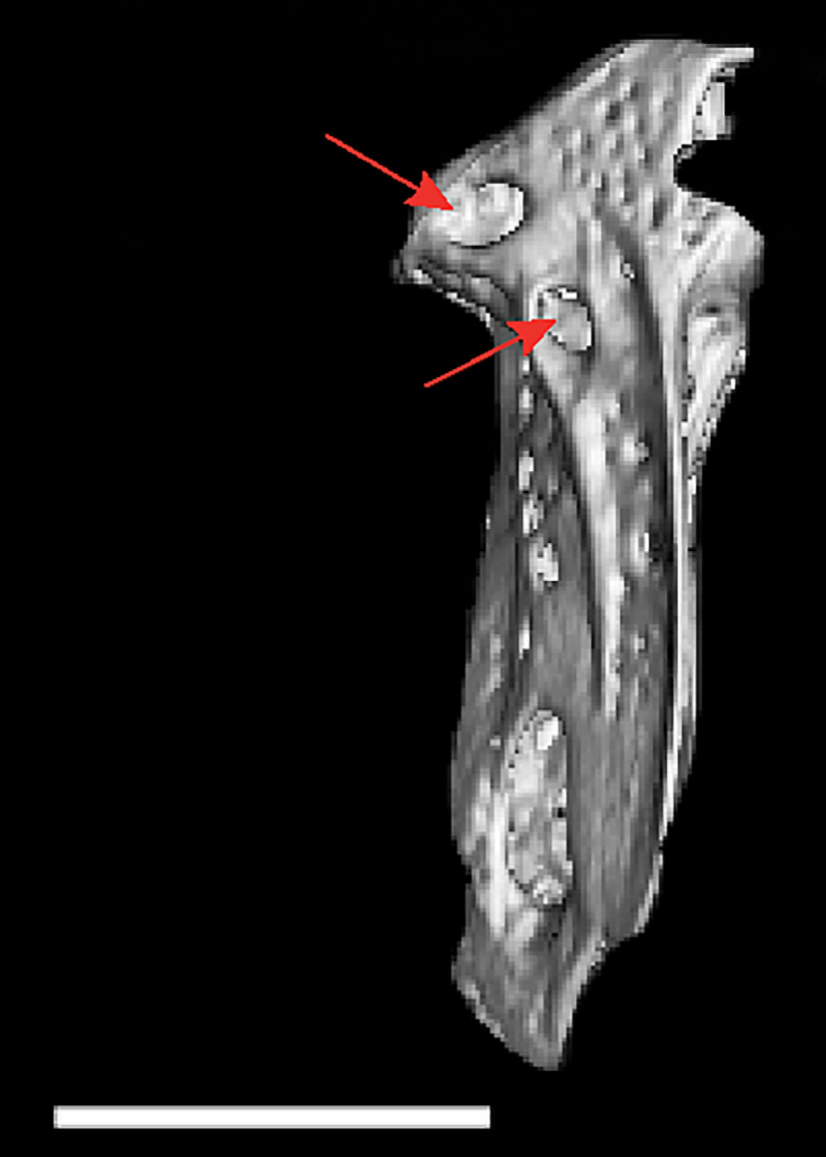
Lateral view of the hyomandibula; arrows pointing two prominent foramina on the dorsal portion of hyomandibula of *Porotergus sambaibensis*, paratype, MZUSP 129865, 98.6 mm length to the end of the anal fin (LEA). Scale bar: 3 mm.

Head and body shape and pigmentation are displayed in Figure 3. Osteological information is provided in Figures 4, 5, 6. Morphometric data are presented in Table 2. Body laterally compressed. Greatest body depth at midpoint of abdominal cavity. Dorsal profile of body nearly straight. Anteriormost perforated scale located above posterior margin of opercle. Head laterally compressed, widest at opercular region. Dorsal profile of head convex. Eye small, circular, completely covered by thin membrane, on anterior one‐half of head length and located laterally on head. Mouth terminal. Rictus extending posteriorly to vertical through posterior margin of eye. Upper and lower jaws equally long. Fleshy lateral lobe of lower jaw small, not visible in ventral view, triangular in shape, located at anterior half of lower jaw and extending beyond vertical through posterior edge of anterior nostril. Anterior nares tubular, closer to snout tip than anterior margin of eye. Posterior naris ovoid, without tubular extension, at anterior third of head.

Branchial opening located slightly anterior to pectoral‐fin origin. Branchial membranes joined at isthmus. Pectoral fin broad and distally pointed with i,14(2), ii,12*(8), ii,13(5) or ii,14(2). Distal pectoral‐fin margin straight, originating beyond anal‐fin origin; fifth or sixth pectoral‐fin ray longest. Anus and urogenital papilla adjacent, located at vertical through posterior margin of eye. Anal‐fin origin at vertical through posterior limit of opercle. Distal margin of anal fin straight. First unbranched anal‐fin rays tiny; rays progressively increasing in size towards first branched rays. Branched anal‐fin rays nearly equally long, except for posteriormost rays that progressively decrease in size. Total anal‐fin rays, 146(1), 147(2), 150(1), 151(3), 152(2), 153*(3), 155(1), 157(1) or 160(2).

Mid‐dorsal body scales from the vertical through the pectoral fin to the dorsal mid‐sagittal electroreceptive filament. Scales cycloid, extending from posterior of head to tip of caudal appendage. Scales present on mid‐dorsal region of body. Lateral‐line scales at vertical through anal‐fin terminus, 60*(1), 61(1), 62(1), 63(3), 64(2), 65(3), 66(1), 67(2) or 68(1). Scales above lateral line, seven (4), eight*(9) or nine (2). Dorsal organ origin on midbody posterior portion at approximately 40% of LEA, structure inserted into narrow mid‐dorsal groove. Elongate, compressed caudal appendage ending in small, lanceolate caudal fin. Caudal‐fin rays, 17(3), 18(1), 19(4), 20(2), 21*(3), 22(2) or 26(1). Scales covering two‐thirds of caudal fin.

### 3.8 Secondary sexual dimorphism

The sex could not be verified as no gonadal differences were found.

### 3.9 Relevant osteological features of *P. sambaibensis*

Frontals surrounding cranial fontanels separate from each other by exposed epiphyseal bar. Anterior fontanel approximately as long as posterior one. Parietal contacting frontal anteriorly, supraoccipital posteriorly and pterotic laterally. Two prominent foramina on dorsal portion of hyomandibula; dorsalmost foramen putative houses anteroventral lateral‐line nerve and ventralmost houses hyomandibular trunk of compound facial nerve. Lateral ethmoid straight positioned.

Premaxilla of moderate size, five (2) conical posteriorly curved teeth. Dentary with 12 (1) or 20 (1) teeth in two rows, with teeth pointing towards mouth cavity, and approximately same length. Ventral ethmoid and vomer ossified. Orbitosphenoid and pterosphenoid well developed. Lateral ethmoid present as ossified tube. Endopterygoid ascending process ossified solely at its base. First branchiostegal ray and second branchiostegal ray absent; third branchiostegal ray slender and located at anterior ceratohyal ventral margin; fourth branchiostegal ray attached to anterior ceratohyal lateral face; fifth branchiostegal ray associated with posterior ceratohyal lateral face. Fourth and fifth branchiostegal rays expanded. Upper pharyngeal tooth plate with seven (1) conical teeth. Fifth ceratobranchial with nine (1) conical teeth.

Precaudal vertebrae 16 (4). Transitional vertebrae three (4). Anterior displaced hemal spine, one*(4); posterior displaced hemal spine, three (6).

### 3.10 Colour in alcohol

Colouration of body dark brown, clearer belly. Pectoral‐ and anal‐fin rays dark brown; interradial membrane hyaline. Caudal peduncle spot present at base of caudal‐fin insertion. Caudal fin dark brown; distal portion hyaline.

### 3.11 Geographical distribution and habitat

**FIGURE 7:**
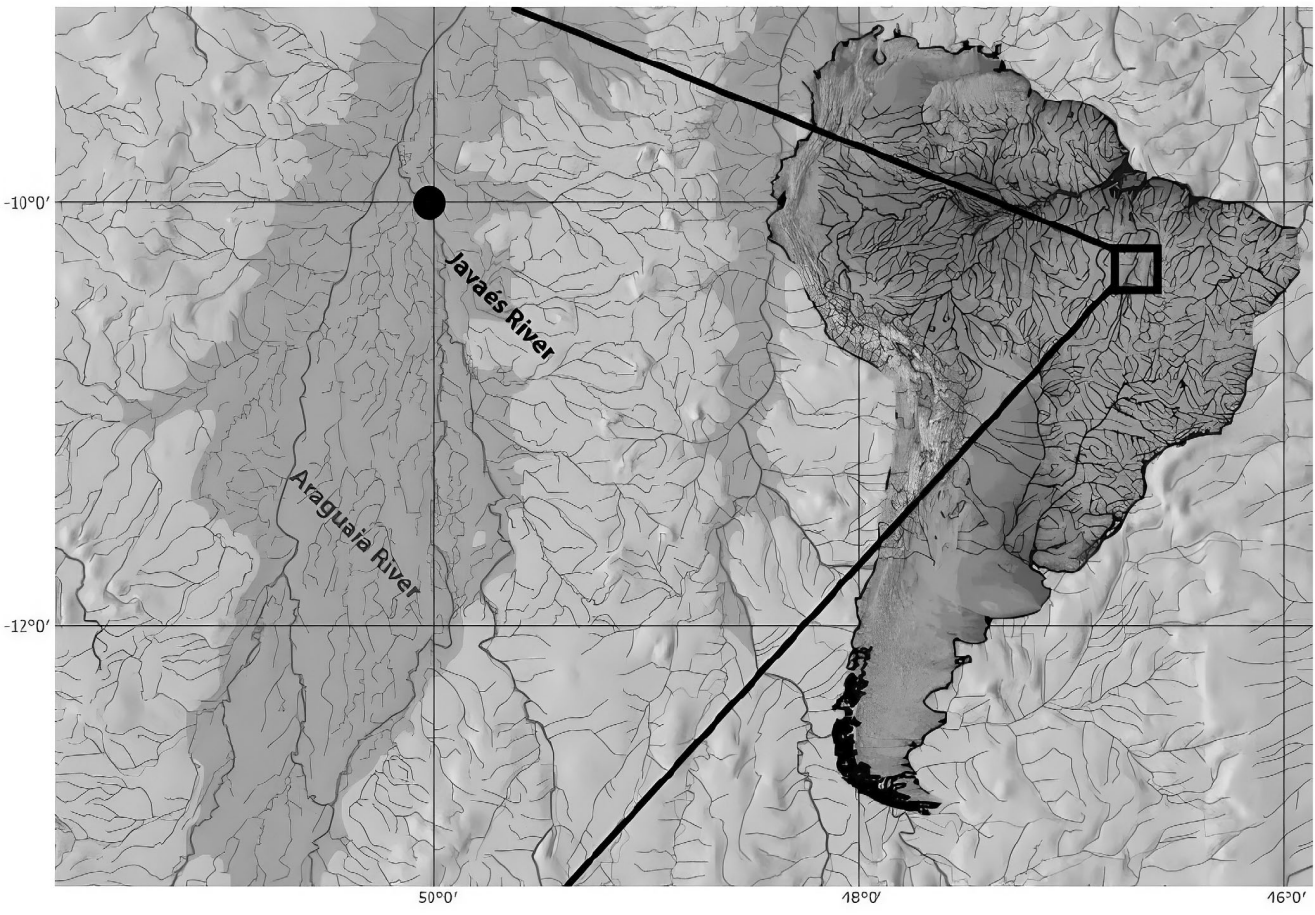
Distribution of *Porotergus sambaibensis*. Black dot represents type series.

*P. sambaibensis* is only known from rocky banks along the Javaés River near Furo do Sambaíba (Figure 7). The rocky banks are composed of lateritic (Chamon et al., 2017: Figure 6), a typical geological formation by the alluvial sedimentation from a median stretch of the Araguaia River (Latrubesse & Stevaux, 2002). The new species was collected with other taxa (Chamon et al., 2017), such as the catfishes (Siluriformes): *Spectracanthicus javae* Chamon, Pereira, Mendonça and Akama 2017, *Auchenipterichthys longimanus* (Günther, 1864), *Ituglanis* sp., *Platydoras armatulus* (Valenciennes, 1840), *Rhamdia quelen* (Quoy & Gaimard, 1824; Pimelodidae) and the cichlids (Cichliformes): *Crenicichla* sp.

### 3.12 Ecological and conservation notes

*P. sambaibensis* is only known from its type locality, the Sambaíba rocky bank in the Javaés River. According to the Geospatial Conservation Assessment Tool (GeoCAT; Bachman et al., 2011), the species would be categorised as critically endangered (CR) due to its restricted extent of occurrence (EO) and area of occupancy (AO). Remarkably, there were no new attempts to collect Gymnotiformes in the Javaés River and its surrounding areas. It is also possible that the newly discovered species can exist in other habitats, like the river channel, as its closely related species have been observed to use different environments during their life cycle. For instance, juveniles of ‘*A*’. *bonapartii* are typically found in macrophyte banks, and mature adults are commonly caught in river channels. Thus, we recommend categorising the species as deficient data (DD) according to the International Union for Conservation of Nature (IUCN) criteria.

The type locality of *P. sambaibensis* is in the middle Araguaia River basin, a place for several protected areas. Although the species' type locality is well protected, increasing anthropogenic activities in the Tocantins‐Araguaia River basin are a potential threat to the region in the medium and long term.

### 3.13 Etymology

The specific epithet is in reference to the type locality, Sambaíba, a rocky bank in the Javaés River.

## 4 DISCUSSION

Based on morphological characters, the monophyly of *Porotergus* and other apteronotids, like *Sternarchogiton*, has been problematic when tested within a phylogenetic framework (de Santana & Crampton, 2010). For example, the characters originally proposed by Ellis in Eigenmann (1912) to define *Porotergus*, including the absence of scales on the dorsal region of the body anterior to the dorsal mid‐sagittal electroreceptive filament; the presence of large scales above the lateral line; the presence of teeth in both jaws; and a long mouth cleft, reaching or passing the vertical through the anterior margin of the eye, are now known to have evolved independently several times in apteronotid (e.g., Bernt et al., 2019; de Santana & Vari, 2010). In the same way, the absence of a pale stripe on the head, the poorly ossified infraorbital series and a maxilla without an anterior shelf used by de Santana and Crampton (2010) to distinguish *Porotergus* within Apteronotidae are ambiguous in a phylogenetic context.

There has been a recent debate regarding whether *P. gymnotus* has scales on the mid‐dorsal body (Bernt et al., 2018), which is the region between the nape and the dorsal mid‐sagittal electroreceptive filament, despite past mentions of their absence (Albert, 2001; de Santana & Crampton, 2010; Ellis, 1912; Mago‐Leccia, 1994). According to Bernt et al. (2019), a larger specimen of *P. gymnotus* (ANSP 189647, 150 mm TL) showed the presence of scales on the mid‐dorsal body. The lot ANSP 189647 originally contained two specimens split into separate lots in 2015 (Mark Sabaj personal communication). Although ANSP 189647 includes an unidentified apteronotid species with scales on the dorsum, ANSP 200961 contains *P. gymnotus*, which does not have scales in the dorsal area. Specifically, two species of *Porotergus* can be found in the upper Maroni River, both represented by one specimen initially in the same lot ANSP 189647 (tissue samples # 6992 and #7021). In 2015, lot ANSP 189647 was split into two lots: ANSP 189647 (#6992) and ANSP 200961 (previously ANSP 189647; #7021). In 2019, Bernt et al. published the sequence (tissue sample #7021) as ANSP 189647. However, as noted here, it belongs to ANSP 200961. As a result, the specimen now in lot ANSP 200961 (formerly ANSP 189647) is tentatively identified as *P. gymnotus* (absence of scales in the entire area between supraoccipital and the dorsal mid‐sagittal electroreceptive filament, and the low number of anal‐fin rays). In contrast, the specimen left in lot ANSP 189647 (scales on the dorsum and high number of anal‐fin rays) is temporally identified as *Porotergus* sp. Consequently, we found no scales on juveniles or adult specimens of *P. gymnotus*, including the type species (e.g., de Santana & Crampton, 2010), voucher specimens in our phylogeny or elsewhere (see examined material). Therefore, all species belonging to the *Porotergus* clade, except ‘*A*’. *bonapartii* and *P. sambaibensis*, do not have scales on the mid‐dorsal body.

Recent studies on the phylogenetic relationships in Apteronotidae, using molecular data (Bernt et al., 2019) and morphological data (Peixoto et al., 2021), recovered the monophyly of *Porotergus* with the inclusion of ‘*A’. bonapartii* species group. Our study corroborated this. Within *Porotergus*, *P. duende* from river channels in the western Amazon evolved as the sister species to all other *Porotergus*. In consecutive order, it was followed by *P. gymnotus* from rapids in the Essequibo and Maroni rivers, ‘*A*’. *apurensis* from the Orinoco River channel (Lasso et al., 2022; Marrero & Taphorn, 1991), a clade with ‘*A*’. *ellisi* from the Paraná River embedded in ‘*A*’. *bonapartii* widespread in Amazon basin river channels (Cox‐Fernandes et al., 2004; Hilton & Cox Fernandes, 2006), and by a clade containing *P. gimbeli*, a typical river channel component in the lowland Amazon basin (Cox‐Fernandes et al., 2004; Crampton, 2007, 2011), and *P. sambaibensis* from rocky banks along the Javaés River. Similar to Bernt et al. (2019), we recovered ‘*A*’. *ellisi* inserted in ‘*A*’. *bonapartii* with a genetic divergence of 0.8%. Therefore, a detailed study on ‘*A*’. *ellisi* taxonomic status is necessary.

In 2016, Tagliacollo and colleagues used molecular and morphological data to establish the monophyly between the *Porotergus* and ‘*A*’. *bonapartii* species group. However, despite high statistical support, no unambiguous anatomical synapomorphy supported this clade. In contrast, Peixoto & de Pinna's, 2022 phylogenetic analysis of Gymnotiformes using myology revealed an autapomorphy that defines *Porotergus* – the origin of *adductor mandibulae* and *pars stegalis*, not including the sphenotic and pterosphenoid. This same condition was found in the new species. As seen here, it is critical to conduct comparative anatomical studies to confidently formulate characters and test synapomorphies hypotheses for *Porotergus* and other Apteronotidae genera (e.g., de Santana & Vari, 2010).

*Porotergus* species, like other Apteronotidae, typically inhabit lowland river channels (Cox‐Fernandes et al., 2004; Crampton, 2007, 2011; de Santana & Crampton, 2010; Marrero & Taphorn, 1991), with occasional invasions of highland habitats, such as rapids, *terra firme* streams and small rivers (de Santana & Vari, 2010). *Porotergus* has undergone two separate habitat transitions. The first change was seen in *P. gymnotus*, which inhabits the Essequibo and Maroni river rapids. The second transition is in *P. sambaibensis*, which lives on rocky banks along the Javaés River in the Brazilian shield.

## 5 MATERIAL EXAMINED

‘*A*’. *apurensis*: MCNG 13878, 18, (110–183 mm); ‘*A*’. *bonapartii*: INPA 13997, 10 (145–225 mm); INPA 4730, 38 (87–172 mm); ‘*A*’. *ellisi*: MZUSP 88263, 15 (214–262 mm); *P. duende*: MCP 37357, holotype, (104 mm); INPA 25257, 1 (CS; 82.4 mm); INPA 24567, 2 (58.5–64 mm); MCP 37359, paratype (69.1 mm); MCP 37360, paratype (84.4 mm); MZUSP 56209, 6 (66–184 mm); MZUSP 56542, 5 (66–107 mm); MZUSP 56544, 2 (134–139 mm); MZUSP 56545, 8 (56–125 mm); MZUSP 57274, 1 (58 mm); MZUSP 57343, 1 (64 mm); MZUSP 57354, 3 (75–115 mm); MZUSP 57975, 1 (79 mm); MZUSP 57976, 1 (94 mm). *P. gimbeli*: FMNH 54566, holotype (200 mm); FMNH 54567, 2 paratypes (166–240 mm); USNM 37308, 19 (106–195 mm); USNM 373007, 8 (117–174 mm). *P. gymnotus*: ANSP 200961, 1 (114 mm); BMNH 1911.10. 31.543, paratype (77 mm); CAS 62305 (ex IU 12636), paratype (85 mm); CAS 31237, 2 (87–102 mm); CAS 72234, 2 (97–113 mm); FMNH 53575 (ex CM 1759), holotype (65.2 mm); FMNH 53291, paratype (57.8 mm); MHNG – GF‐510‐13, 4 (57–107 mm). *Porotergus* sp.: ANSP 189647, 1 (146 mm).

## AUTHOR CONTRIBUTIONS

Marina B. Mendonça: contributed substantially to the conception and design of the study; the acquisition, analysis and interpretation of data; and the writing of the manuscript. Luiz A. W. Peixoto: contributed substantially to the acquisition, analysis and interpretation of data, as well as the writing of the manuscript. Carine C. Chamon: contributed substantially to the conception and design of the study and the writing of the manuscript. A. Akama: contributed substantially to the conception and design of the study and the writing of the manuscript. C. David de Santana: contributed substantially to the conception and design of the study; the acquisition, analysis and interpretation of data; and the writing of the manuscript.

## FUNDING INFORMATION

The Discovery of the initial specimens was made possible by the expedition funding provided by the National Research Program on Biodiversity MPEG/FADESP (No. PPBio project 3570). This work was partially developed at the Molecular Biology Laboratory of the MPEG with resources from FINEp for the project “Analytical Park of the MPEG: analysis of the transformations of the Amazon and its effects on socio‐biodiversity and the landscape” (No. 0118003100), coordinated by ALCP.CDS and LAWP were funded by FAPESP (No. 2016/19075‐9). LAWP was supported by Conselho Nacional de Desenvolvimento Científico e Tecnológico (No. CNPq#168395/2022‐3); Fundação Amazônia de Amparo à Estudos e Pesquisas (No. FAPESPA#2023/158693); São Paulo Research Foundation under Grant (No. FAPESP #2018/05084‐1).

## Supporting information


**Figure S1.** Relationships in Apteronotidae from maximum likelihood reconstruction of the concatenated matrix for 1000 bootstrap replicates. Green: *Porotergus sambaibensis*; dark blue: *Porotergus gimbeli*; purple: ‘*Apteronotus*’ *bonapartii* + ‘*Apteronotus*’ *ellisi*; orange: ‘*Apteronotus*’ *apurensis*; red: *Porotergus gymnotus*; light blue: *Porotergus duende*.
