# Supplementary figures and images for: Molecular phylogeny reveals a new species of ghost electric knifefish Porotergus Ellis 1912 (Gymnotiformes: Apteronotidae), from the Amazon basin
Source: J Fish Biol. 2025 Jul 14;107(5):1519–31. doi: 10.1111/jfb.70085 (PMC12710834; doi:10.1111/jfb.70085)

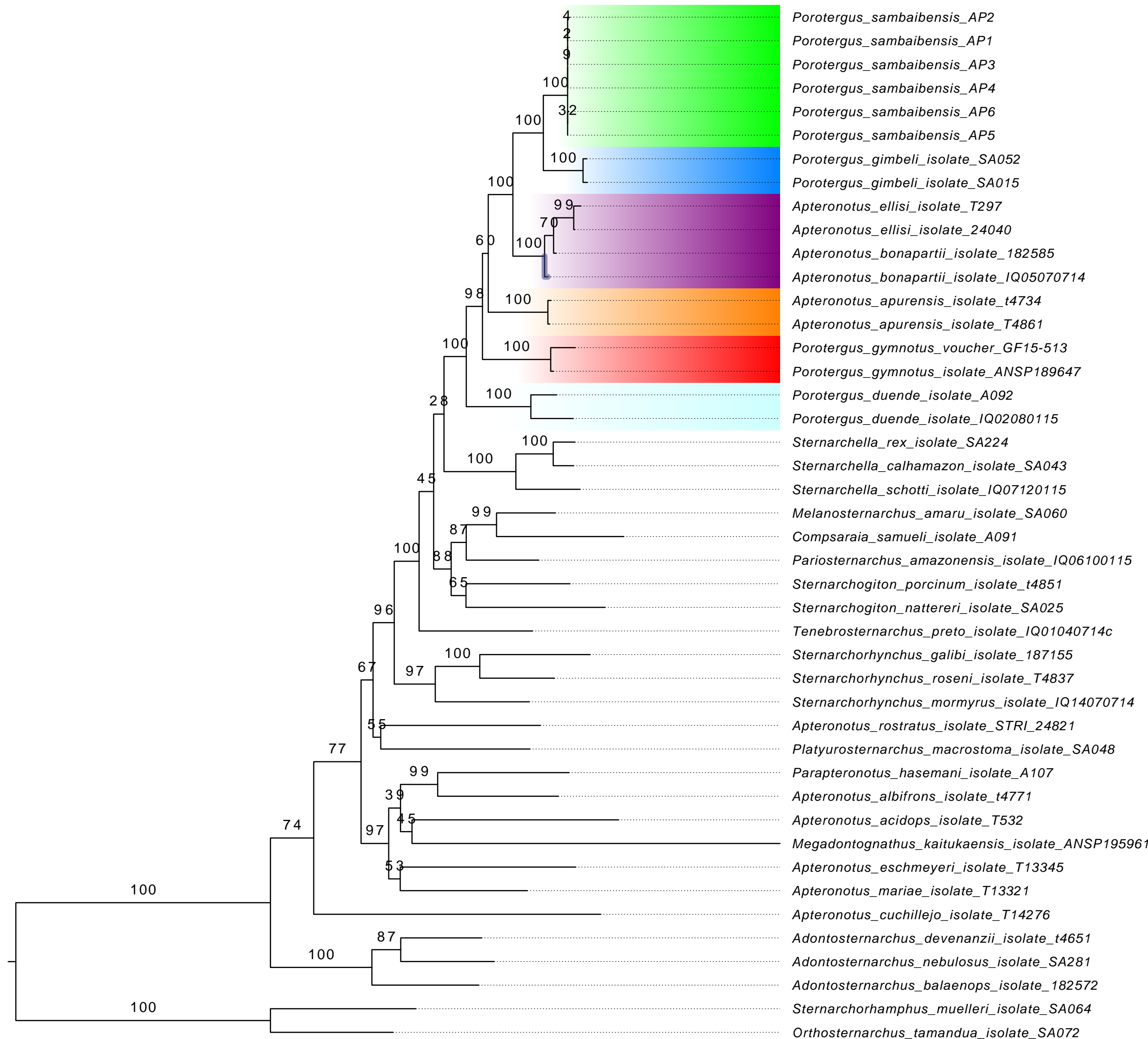

Supplement: Supplementary file 1 — Figure S1. Relationships in Apteronotidae from maximum likelihood reconstruction of the concatenated matrix for 1000 bootstrap replicates. Green: Porotergus sambaibensis; dark blue: Porotergus gimbeli; purple: ‘Apteronotus’ bonapartii + ‘Apteronotus’ ellisi; orange: ‘Apteronotus’ apurensis; red: Porotergus gymnotus; light blue: Porotergus duende. [file JFB-107-1519-s001.pdf]
